# Comparative Genomics of *Clostridium baratii* Reveals Strain-Level Diversity in Toxin Abundance

**DOI:** 10.3390/microorganisms10020213

**Published:** 2022-01-20

**Authors:** Claudia Silva-Andrade, Alberto J. Martin, Daniel Garrido

**Affiliations:** 1 Laboratorio de Biología de Redes, Centro de Genómica y Bioinformática, Facultad de Ciencias, Universidad Mayor, Santiago 8580000, Chile; claudia.silvaa@mayor.cl; 2 Department of Chemical and Bioprocess Engineering, School of Engineering, Pontificia Universidad Católica de Chile, Santiago 7820436, Chile

**Keywords:** *Clostridium*, pangenome, MLSA, botulism toxin, phylogenetics

## Abstract

*Clostridium baratii* strains are rare opportunistic pathogens associated with botulism intoxication. They have been isolated from foods, soil and be carried asymptomatically or cause botulism outbreaks. Is not taxonomically related to *Clostridium botulinum*, but some strains are equipped with BoNT/F7 cluster. Despite their relationship with diseases, our knowledge regarding the genomic features and phylogenetic characteristics is limited. We analyzed the pangenome of *C. baratii* to understand the diversity and genomic features of this species. We compared existing genomes in public databases, metagenomes, and one newly sequenced strain isolated from an asymptomatic subject. The pangenome was open, indicating it comprises genetically diverse organisms. The core genome contained 28.49% of the total genes of the pangenome. Profiling virulence factors confirmed the presence of phospholipase C in some strains, a toxin capable of disrupting eukaryotic cell membranes. Furthermore, the genomic analysis indicated significant horizontal gene transfer (HGT) events as defined by the presence of prophage genomes. Seven strains were equipped with BoNT/F7 cluster. The active site was conserved in all strains, identifying a missing 7-aa region upstream of the active site in *C. baratii* genomes. This analysis could be important to advance our knowledge regarding opportunistic clostridia and better understand their contribution to disease.

## 1. Introduction

Species belonging to the Firmicutes phylum are the most numerous in the gut microbiota, with several *Clostridium* species among the most characterized and studied of its members [1]. *Clostridium* is an important genus of Gram-positive, obligate anaerobes, bar-shaped spore-forming rods. *Clostridium* species could be found in soil and residing in several animals’ intestines, showing their widespread distribution [2]. This taxonomic group includes important animal and human pathogens that cause dangerous and potentially deadly diseases such as tetanus [3] (produced by tetanus toxin from *C. tetani*), gastroenteritis [4] (*C. perfringens*), *Clostridioides difficile* infection, and botulism [5,6] (produced by botulism neurotoxin from *C. botulinum*), among several others [7]. In addition to their medical importance, *Clostridium* species are well studied for their industrial properties in producing important bioproducts such as ethanol, organic acids, and therapeutic proteins [8].

Interestingly, some members of the genus seem to be essential in the establishment of the immune tolerance to the gut microbiota, an effect mediated in part by regulatory T-cells and butyrate as a fermentation end-product [9]. Taxonomically, *Clostridium* sensu stricto species belong to the *Clostridium* Cluster I, based on 16S rRNA gene sequences, according to Collins et al. [10]. This group includes *C. botulinum* and other less-studied species such as *Clostridium baratii.*

*C. baratii* strains are Gram-positive rods, non-motile spore-forming bacteria. *C. baratii* is not taxonomically related to *C. botulinum*, but some members of this species have caused botulism outbreaks associated with food consumption, as well as sporadic infant botulism cases (less than 1% of total cases). These outbreaks are described as sporadic and rare episodes [11,12,13,14,15]; however, the strains that possess the neurotoxin may pose a significant threat, and it is essential to further study the properties of these microorganisms. *C. baratii* strains have been found in liver pastes, meat, soil, and other foods. The species is considered an opportunistic pathogen that can cause disease in subjects with altered gut microbiomes or undergoing antibiotherapy, both infants and adults.

Botulism intoxication is characterized by flaccid paralysis due to the blocking of neurotransmitter release (usually acetylcholine) at the neuromuscular junction. There are eight serotypes of BoNT toxins (A–G, and X) [16], which differ in their protein targets [17]. The *C. baratii* isolates responsible for the outbreaks have been shown to carry the botulinum neurotoxin (BoNT) and its gene cluster responsible for the production of the Type F botulinum toxin [12,13,14,15,16,18,19,20,21,22,23]. The F serotype is generally produced by *C. botulinum* strains and some strains of *C. baratii.* There are nine subtypes of the botulinum toxin F serotype [24]. These subtypes exhibit unique catalytic properties, sequence diversity, and substrate specificities [21,24]. The BoNT gene forms a cluster including UviA, UviB, ORF-X3, ORF-X2, ORF-X1, p47, NTNH, and BoNT genes [13]. The non-toxic non-hemagglutinin (NTNH) interacts directly with BoNT and plays an important role in protecting the toxin in the gastrointestinal environment, facilitating its cellular transport and release [25]. The UviA gene appears to perform a regulatory role similar to BotR in other serotypes [18].

*Clostridium* species play a pivotal and intricate role in the gut microbiota. Under normal circumstances, they promote gut homeostasis and contribute to the barrier effect and balance in mucosal immunity [26,27]. However, several members produce a wide array of toxins and contribute to severe diseases in certain conditions. In this study, we aimed to determine relevant genomic determinants of *C. baratii* genomes to better understand their diversity and their contribution to disease. To do so, we compared and studied all available *C. baratii* genomes, including one derived from a new isolate obtained by us, focusing on the identification of genes associated with the pathogenicity of this species.

## 2. Materials and Methods

### 2.1. DNA Isolation, Library Preparation, and Illumina Sequencing

*Clostridium baratii* C3 strain was isolated from fecal samples from a healthy subject in a previous study [28]. The strain was isolated using a YCFA medium [29] after three rounds of purification. Bacteria were cultured in Reinforced *Clostridium* Medium under anaerobic conditions in an anaerobic jar (Anaerocult), and genomic DNA was extracted using a modified phenol-chloroform protocol. DNA was submitted to the sequencing service MicrobesNG (Birmingham, UK) for sequencing using Illumina MiSeq with a minimum coverage of 30×, and 250 bp pair-end reads.

### 2.2. De Novo Assembly and Functional Annotation

Reads were trimmed with Trimmomatic [30]. Trimmed reads were assembled into contigs using the next genome assemblers: Mira [31], MaSuRCa [32], and SPAdes [33]. Each assembler was executed with default parameters. Finally, the result of the three assembly tools was merged with the option untrusted-contigs of SPAdes. Prodigal [34] was used to identify predicted open reading frames (ORFs) in all contigs from assembly genomes. Assembly contigs were annotated using BLAST+ [35] for Clusters of Orthologous Groups (COG) [36], Carbohydrate-Active enZYmes (CAZy) [37], TransportDB [38,39], Victors [40], and VFDB [41,42] databases and using HMMER [43] v3.1b2 against Pfam [44] database as available on July, 2020. Following, eggNOG-mapper [45] was used for annotation through orthology assignment. Finally, Interproscan [46] was used with default parameters to assign functional annotations based on sequence homology detection.

### 2.3. Genomes

The genome of our isolated *C. baratii* strain was used to perform comparative genomics with the existent genomes available in the Reference sequence (RefSeq) database at NCBI available on September 2021 [47]. The data used for comparative genome analysis are described in Supplementary Table S1. The data used for Multi Locus Sequence Analysis were the genomes used in Kiu et al. [7].

### 2.4. Multi Locus Sequence Analysis (MLSA)

To obtain a better resolution of the phylogenetic relationships of our isolated *C. baratii* strain, we included in our analysis all *Clostridium* pathogenic strains used in Kiu et al. [7]. Then performed a Multi Locus sequence analysis [48] with the 15 ribosomal protein data sets (ribosomal proteins L2, L3, L4, L5, L6, L14, L16, L18, L22, L24, S3, S8, S10, S17, and S19) used in Hug et al. [49]. We used Orthofinder [50] to search orthologous genes between the strains, followed by RaxML [51], a tool that calculates the phylogenetic distance and creates a Maximum Likelihood phylogenetic tree. Finally, the phylogenetic tree was visualized with Figtree [52].

### 2.5. Comparative Genome Analysis

Homologous groups of protein-coding genes from multiple bacterial genomes were built using Roary [53]. Roary was run with the following parameters: “roary-p 10-r-e-i 90-f./data/*.gff”. We visualized the output of Roary with roary_plots.py and create_pan_genome_plots.R scripts, provided with the software.

We employed the reference pangenome created by Roary as an input to EggNOG 4.5.1 eggNOG-mapper v2 genome-wide functional annotation tool [45], ran default parameters, and the DIAMOND [54] mapping mode.

We evaluate the Clusters of Orthologous Groups (COGs) [36] found in the pangenome, sorting the results from EggNOG by COG categories. The values associated with the COG categories represent the percentage belonging to the core genome out of the total COG found. Each category is counted independently.

We used a blastp search against the Carbohydrate-Active Enzymes (CaZy) [37] Database Available online: http://www.cazy.org/ (accessed on 5 January 2021) to identify genes of carbohydrate metabolism. CaZy hits were considered positive if they had a 60% identity and coverage in blast result.

Genes related to the GH (glycoside hydrolases) CaZy category in the pangenome were extracted from each strain using in-house Perl and bash scripts. Bar plots were generated using the R ggplot2 package [55].

### 2.6. Genomic Analysis

To identify the virulence potentials, we performed an in silico analysis of 29 identified toxins and virulent enzyme sequence data found in different *Clostridium* strains from the Virulence Factor Database (VFDB, as available on July 2020) [41,42]. To identity prophage profiles, we used the online tool PHASTER [56,57] (PHAge Search Tool Enhanced Release). To identify antimicrobial resistance, we download the sequences from the Comprehensive Antimicrobial Resistance Database [58] (CARD), as available in January 2021. These sequences were used to build a BLAST database.

To identify the plasmid in *C. baratii* strains, we performed an in silico analysis using the Comprehensive Database of Plasmid Sequences [59]. With the result of plasmid, we searched the BoNT/F7 cluster and evaluated the proteins from the plasmid present in *C. baratii* strains with BLAST+ [35].

BLAST+ [35] was employed for sequence similarity search (blastP) using thresholds for percentage identity and coverage of 80% and e value of 1 × 10^−5^ were applied. Heat maps were generated using the R [55] pheatmap package.

BoNT gene clusters were downloaded from the NCBI database from the code JX847735 and used to build a BLAST database. BLAST+ was employed for sequence similarity search (blastP) using thresholds for percentage identity and coverage of 60% and e value of 1 × 10^−5^. Heat maps were generated using the R pheatmap package. To understand the phylogenetic relationship between the BoNT genes in *C. baratii* and *C. botulinum* strains, we used RAxML [51] to calculate the phylogenetic distance and create a Maximum Likelihood phylogenetic tree with all sequences of BoNT genes. Finally, the phylogenetic tree was visualized with Figtree [52].

To evaluate the possible effect on the three-dimensional structure of the loss of seven amino acids upstream of the active site in BoNT toxin in *C. baratii* genomes, we used the BoNT/F7 gene from AGR53840.1 and performed a tertiary structure prediction with Phyre2 [60]^.^ We compared this gene to the known three-dimensional structure of BoNT/A1 (PDB: 3BTA) in VMD [61] using Multiseq tools to align the structure of both proteins.

### 2.7. Microbiological Assays

*C. baratii* C3 was routinely cultured in Reinforced *Clostridium* Medium (RCM, Becton-Dickinson, Franklin Lakes, NJ, USA) supplemented with 0.5 g/L of L-cysteine (RCM-cys; Loba Chemie, India). All incubations were performed at 37 °C for 24–48 h in an anaerobic jar (Anaerocult, Merck, Darmstadt, Germany) with anaerobic packs (Gaspak EM, Becton-Dickinson, Franklin Lakes, NJ, USA). Two overnight cultures of *C. baratii* were made on Blood Agar and Starch Agar plates (Winkler, Santiago, Chile). The bacteria were also inoculated in API-20A strips (Biomerieux, Craponne, France) following the manufacturer’s instructions. Finally, antibiotic resistance patterns were evaluated using antibiotic discs in RCM agar plates incubated for 48 h (Lilfilchem, Roseto degli Abruzzi TE, Italy) for 48 h. Cefazolin (30 µg), ciprofloxacin (5 µg), chloramphenicol (30 µg), erythromycin (15 µg), penicillin G (10 µg), piperacill/tazobactam (110 µg), trimethoprim-sulfamethoxazole (25 µg), and vancomycin (30 µg) were used.

## 3. Results

### 3.1. General Features

Our analysis included fourteen public genomes of *C. baratii* available from IMG [62,63,64] and the Reference sequence (RefSeq) database. Genomes were obtained from single species or public metagenomes [47,65], and a novel strain isolated from an asymptomatic subject fecal samples, *C. baratii* C3. In publicly accessible metagenomes studies from the NCBI [66] database, we observed that the frequency of finding *C. baratii* genomes across human gut metagenomes was very low: from 2588 human gut meta-genome bioprojects only four contained *C. baratii* genomes (Supplementary Table S1).

On average, using the fifteen genomes employed in this work, the genome size of *C. baratii* was 3,102,724 bp and it contained 2911 predicted protein-coding genes (Supplementary Table S1). The genome size of *C. baratii* C3 was 3,119,424 bp with 2972 predicted protein-coding genes, similar to other *C. baratii* genomes.

### 3.2. Probing Evolutionary Relationships between Clostridium Strains

**Figure 1:**
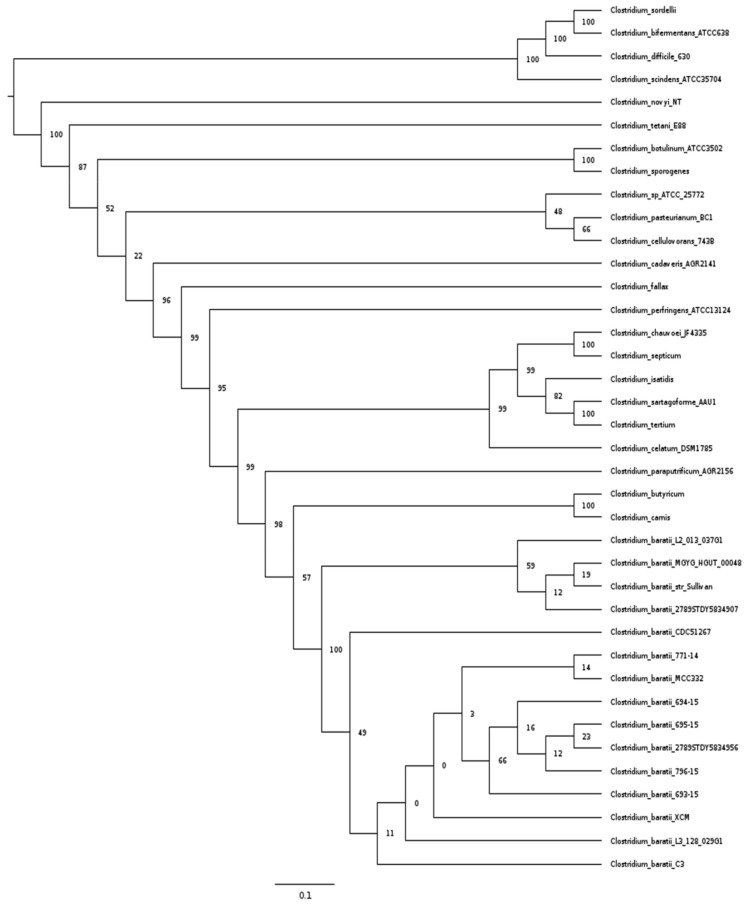
MLSA analysis of pathogenic *Clostridium* strains. Maximum Likelihood phylogenetic tree of pathogenic *Clostridium* strain based on 15 ribosomal protein data sets.

We used the multi-locus sequence analysis approach (MLSA [48]) to understand the phylogenetic relationship between the fifteen genomes of *C. baratii* described above and the pathogenic clostridia used in Kiu et al. [7]. The pathogenic clostridia used in this study and toxins or diseases that produce are listed in Supplementary Table S2 This analysis showed that all *C. baratii* strains formed a monophyletic lineage in the phylogenetic tree, and the closest relative genomes were found in a cluster containing *Clostridium carnis* and *Clostridium butyricum* genomes [67]. *C. baratii* C3 appeared to be closely related to a monophyletic lineage in the phylogenetic tree formed by *C. baratii* strains L3_128_029G1, XCM, 693-15, 796-15, 2789STDY5834956, 695-15, 694-15, MCC332, and 771-14 (Figure 1).

### 3.3. Comparative Genomics Analysis

**Figure 2:**
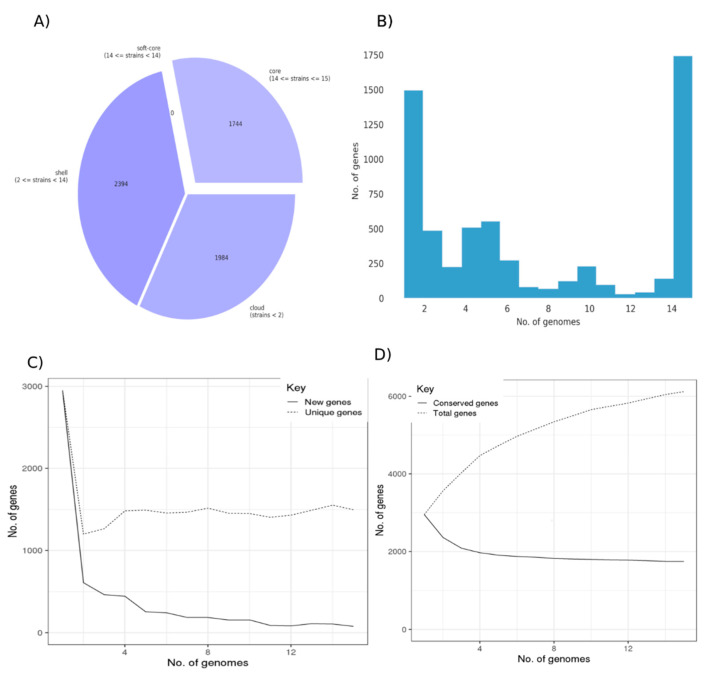
Pangenome of *Clostridium baratii* strains. (**A**) Core and accessory genes statistics. (**B**) Frequency bar graphs of the number of identical genes against the number of genomes. (**C**) Number of new genes and unique genes in the pangenome as genomes are included. (**D**) Number of conserved genes and total genes as more genomes are included in the pangenome calculation.

To identify variable and conserved functions in *C. baratii*, we constructed the pangenome of this species from all fifteen available genomes. The pangenome of *C. baratii* comprised 6122 genes, 1744 core genes, and 4378 accessory genes (Figure 2A). Remarkably, 28.49% of the pangenome represented core genes, and 24.43% (1.496 genes) were unique (defined as genes present only in one strain; Figure 2A–C). This analysis indicated that *C. baratii* comprises genetically diverse organisms since it has a highly variable pangenome (Figure 2D). Given available genomes, we estimate that new genes could potentially be added at an average of 226 genes for each new genome sequenced (Supplementary Figure S1).

### 3.4. COG Analysis

**Figure 3:**
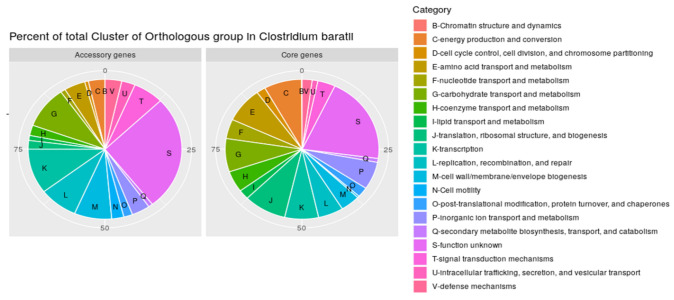
Percent of total clusters of orthologous groups (COGs) in pangenome. Percentage of total Cluster of Orthologous group annotated in the *Clostridium baratii* core genome and accessory genes.

We then analyzed the functions of the core and accessory genes of the *C. baratii* pangenome by assigning them to “Clusters of Orthologous Groups” (COG) database [36]. Genes with unknown functions were more abundant among accessory genes than in the core pangenome (332 core genes vs. 790 accessory genes). Interestingly, 242 accessory genes were assigned to a functional cluster associated with “replication, recombination and repair” (“L” COG code) versus 88 core genes (Figure 3), the majority of genes from accessory genes in this category were associated with transposases, integrases, and phages. This difference suggests that *C. baratii* genomes have variations in their repair mechanisms and adaptations to stress. Similarly, genes associated with cell wall/membrane/envelope biogenesis (“M” COG code) were found more often in the accessory pangenome (233 genes) than in the core genes (68 genes). Finally, the number of genes associated with carbohydrate transport and metabolism (“G” COG code) was higher among accessory genes than in core genes (accessory: 288 genes vs. core: 127 genes). This suggests that carbohydrate utilization functions are conserved among strains of *C. baratii.*

### 3.5. Carbohydrate-Active Enzyme Analysis

**Figure 4:**
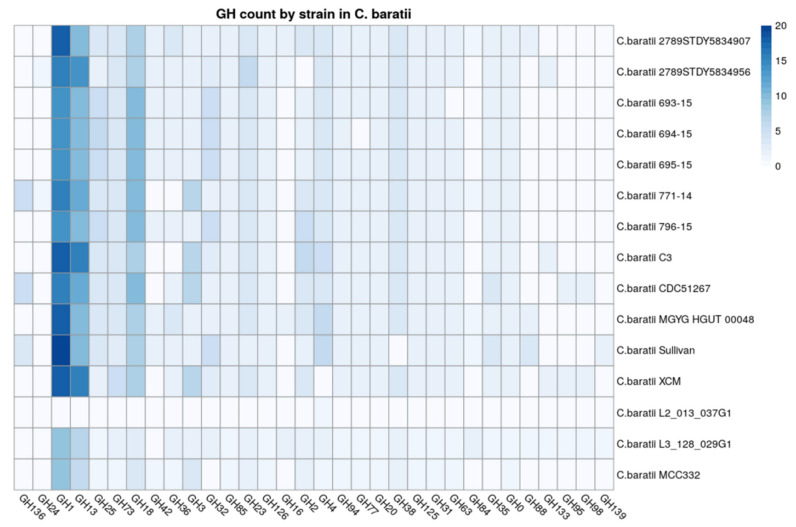
Count of Glycosyl Transferases (GH) categories identified in strains. GHs in *Clostridium baratii* genomes. The x-axis represents the count of the Glycosyl Transferases category, and the y-axis represents the strains.

Glycoside hydrolases were identified as the enzyme family with more members versus other carbohydrate-active enzymes among *C. baratii* genomes (Supplementary Figure S2). We found that the GH1 family was the most represented in *C. baratii* strains (Figure 4). Among GH1, 21.43% were predicted as β-galactosidases according to their EC numbers, and 78.57% were predicted as β-glucosidases. The second most represented family was GH13, a family represented by α-amylases. Their abundance indicates a conserved starch-utilization capability among *C. baratii*, especially strains C3 and XCM. The third most represented family was GH18, known as hexosaminidases releasing N-acetyl-β-D-glucosaminide/galactosaminide linkages in host-derived glycans and chitins. This observation suggests a limited ability to metabolize host-derived glycans, considering the absence of other important GH families including sialidases and fucosidases.

### 3.6. Virulence Factors

**Figure 5:**
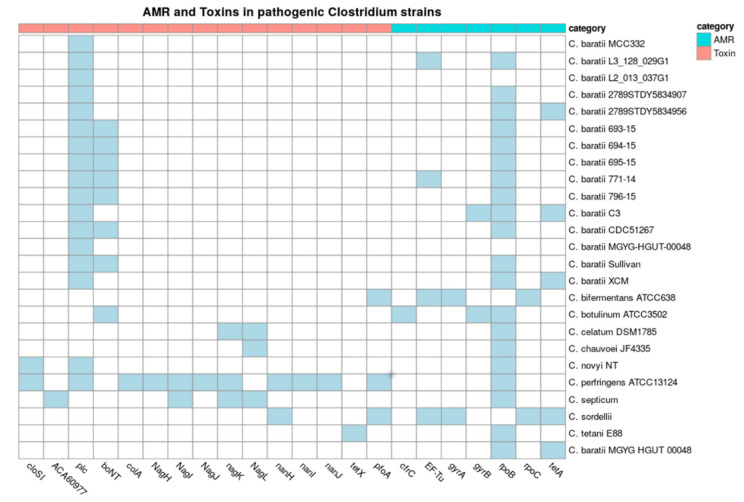
Toxins and Antimicrobial resistance (AMR) in pathogenic *Clostridium* strains. Presence (in blue) or absence (in white) of toxin and AMR genes in pathogen *Clostridium, C. baratii* strains. The x-axis represents different toxins, and the y-axis represents the strains. The color on heatmaps represents the toxin group by category.

We later examined the presence of virulence factors among all analyzed *C. baratii* genomes and other bacteria in the *Clostridium* genus that are known to cause diseases to be pathological (Figure 5). The BoNT gene F7 serotype, encoding the botulinum toxin, was present in seven out of fifteen genomes of *C. baratii*, being absent in strains MGYG-HGUT-00064, 2789STDY5834956, 2789STDY5834907, XCM, L3_128_029G1, MCC332, L2_013_037G1, and C3. The membrane-active alpha-toxin phospholipase C (plc) was present in all *C. baratii* genomes (Supplementary Table S3). *C. perfringens* ATCC 13,124 contained the most diverse repertoire of toxin genes, including plc, sialidases (nanH, nanI, and nanJ), theta-toxin/perfringolysin O (pfoA), mu-toxin (NagH, NagI, NagJ, nagK), and microbial collagenase (colA).

Additionally, we evaluated the presence of antimicrobial resistance genes (AMR) and phage content among these *Clostridium* genomes (Figure 5). All but two clostridial pathogens and three *C. baratii* strains had a rpoB gene (CARD code ARO:3004563) with point mutations conferring putative resistance to rifampin and rifampicin. The genome of *C. sordelli* contains the most diverse repertoire of AMR genes, including EF-Tu mutations (CARD code ARO:3003357) conferring resistance to elfamycin, gyrA (fluoroquinolones), rpoC (rifampicin), tetA, and tetB (tetracycline resistance). *C. baratii* strains 2789STDY5834956, C3, and MGYG-HGUT-00064 had a tetA gene. Strain C3 carried point mutations in gyrB (CARD code ARO:3004562) with point mutations conferring resistance to fluoroquinolone antibiotics. Strain XCM carried tetA and tetB genes (Figure 5). These results indicate that certain *C. baratii* strains could carry relevant AMR genes. However, they were scattered among *C. baratii* genomes.

Finally, genome scanning for prophage elements revealed that phage phiSM101 and vB_Cpes_CP51, bacteriophages that belong to the Siphoviridae family. The Siphoviridae family is a diverse family that infects the Enterobacteriaceae bacterial family and carries virulence genes acquired by other microorganisms [68]; they are present in *C. perfringens* [25] and only present in *C. baratii* strains 771-14, XCM, Sullivan 2789STDY5834956, and 2789STDY5834907. The PHASTER Putative prophages analysis showed that all *C. baratii* genomes except strain 695-15 contain several prophage proteins, ranging from eight to more than a hundred (Supplementary Table S4).

Plasmids were found only in Sullivan and CDC51267 strains, which have finished genomes sequences. *C. baratii* strain l Sullivan carries the plasmid pCBJ, with a size of 185,364 bp and 212 coding regions [18], and *C. baratii* strain CDC51267 carries the plasmid pNPD11_1, a circular plasmid with 119 coding regions and a size of 120,667 bp [69]. Both strains have the BoNT/F7 cluster in the plasmid. We found evidence of protein from plasmids pNPD11_1 in strains 693-15, 694-15, 695-15, 771-14, and 796-15, (Supplementary Table S5)

### 3.7. Phenotypical Assays of C. baratii C3

**Figure 6:**
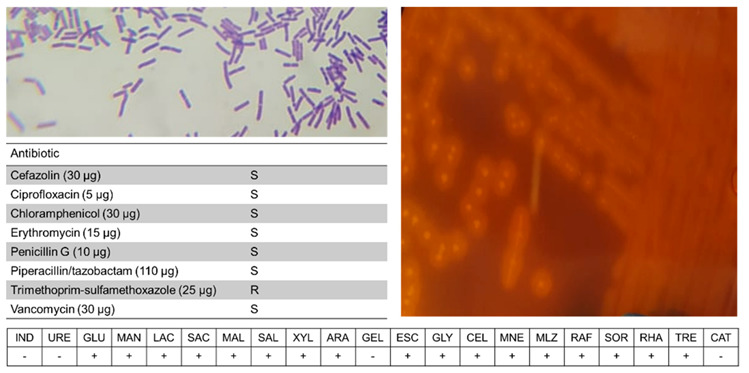
Phenotypical analysis of *C. baratii* C3. Shown are Gram-staining, blood agar analysis, disk diffusion, and API-20A results.

Strain C3 was shown to have a β-hemolytic activity revealing the presence of exotoxins, and positive for starch degradation, indicating amylase activity (Figure 6). Biochemical tests indicated several features in common with other *C. baratii* strains, but compared to other studies C3 was unusual in the utilization of xylose, arabinose, melezitose, and raffinose. Disk diffusion tests indicated that C3 was resistant to trimethoprim-sulfamethoxazole.

### 3.8. Distribution of the BoNT Gene Cluster in C. baratii

**Figure 7:**
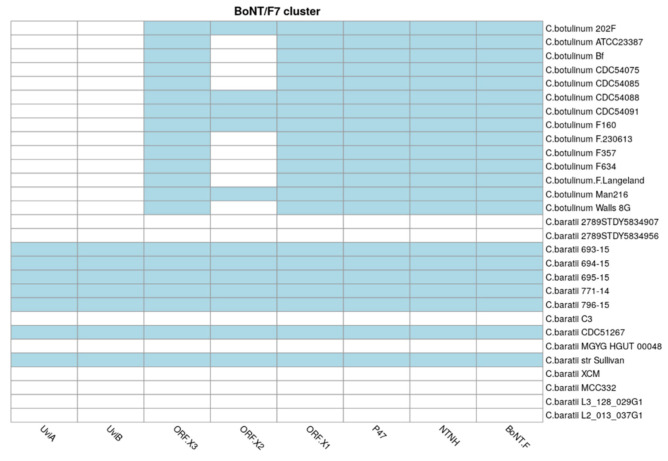
BoNT toxin cluster. Presence (in blue) or absence (in white) of BoNT toxin cluster genes in *C. baratii* and *C. botulinum* strains. The x-axis represents BoNT cluster genes, and the y-axis represents the strains.

We finally studied the distribution and diversity of the BoNT gene cluster in this species. *C. baratii* strains 693-15, 694-15, 695-15, 771-14, 796-15, Sullivan, and CDC51267 carried the BoNT/F cluster, while it was absent in the other eight genomes studied (Figure 7). The proteins encoded by genes in this cluster, represented by genes BoNT/F, NTNH, UviA, UviB, P47, ORF-X1, ORF-X2, and ORF-X3, synthesize the BoNT/F toxin. *C. baratii* 693-15, 694-15, 695-15, 771-14, 796-15, CDC51267 and Sullivan strains showed an organization and gene cluster contents identical to serotype E and similar to serotype F6, which do not contain the botR gene. Serotype F6 differs from F7 and E as it contains an is element between the orfX3 and orfX2 genes. The proteolytic clostridia F1, F2, F4, and F5 contain botR [18].

The NTNH gene was also found in all *C. baratii* genomes carrying the BoNT cluster, as well as UviA and UviB, suggesting that the UviA/B protein complex participates in the regulation of the production of botulinum neurotoxin [18] in *C. baratii*, similar to other *Clostridium* pathogens [70,71], but this remains to be shown experimentally.

At the amino acid sequence level, the BoNT gene clustered separately between *C. baratii* and *C. botulinum* genomes (Supplementary Figure S3). BoNT sequences were well conserved among *C. baratii* genomes that contained the gene set. The BoNT proteins from *C. baratii* strains had on average a 72% identity sequence with the BoNT/F from *C. botulinum* strains (Supplementary Figure S3).

**Figure 8:**
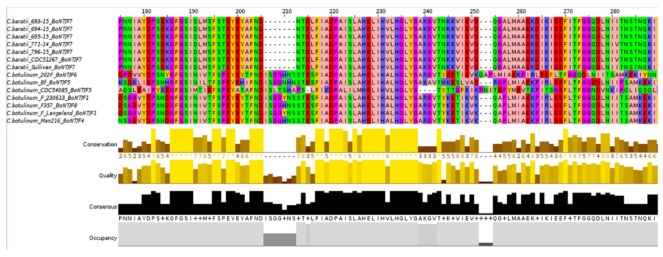
BoNT gene alignment. Alignment of BoNT gene in *C. baratii* and *C. botulinum* strains. *C. baratii* strains do not have 7 amino acids present in the BoNT gene of *C. botulinum* in position 204, and the active site of the type botulinum toxin is the HEXXH sequence, located in positions 211–215.

The active site of the type botulinum toxin is the HEXXH sequence, located in positions 211–215 [72,73]. This motif and surrounding positions were well conserved among all *C. baratii* and *C. botulinum* strains (HELIH sequence). Only a 7-aa region upstream of the active site appeared to be missing across *C. baratii* genomes compared with *C. botulinum* strains (Figure 8). It has been shown that BoNT/F from *C. baratii* can cleave VAMP at the same site as the other BoNT/F serotypes [74,75,76]. Therefore, the 7 amino acids gap present in *C. baratii* genomes is not likely to affect the activity of the toxin and its production since this gap is located far from the active site of the protein described in Breidenbach et al. [77] (Supplementary Figure S4).

## 4. Discussion

The species *C. baratii* includes strains that are known to be responsible for botulism outbreaks, a dangerous form of food poisoning [15,19,23]. The BoNT serotype F has been associated with outbreaks caused by the consumption of meat-derived foods [13,78]. However, our knowledge of the diversity and genome features of this species is limited. Here, we compared the fifteen genomes of *C. baratii*, including two obtained from metagenomes and the genome of a strain we previously isolated from an asymptomatic subject. Unfortunately, the availability of genome sequences from outbreaks and clinical cases is very low, limiting our analysis to a reduced set of genomes. In addition, the isolation of a neurotoxigenic organism from feces does not necessarily imply that living bacteria have colonized the intestinal area since inert spores passing through the intestinal tract may be the source of the isolate. However, it is uncertain if this applies to *C. baratii* since it has been found in different environments.

*C. baratii* appears to be a very infrequent species in the gut microbiota (Supplementary Table S1). *C. baratii* genomes form a monophyletic lineage, not phylogenetically related to any known pathogen clostridia. The comparative analysis shows an open pangenome, indicating that more genomes of this species are needed to understand its full genomic diversity. This observation is in the same line as previous studies indicating *C. baratii* represents genetically diverse organisms [3,7]. Similarly, several other pathogenic microorganisms have open pangenomes, including *C. perfringens* [7] and *Legionella pneumophila* [79]. Different bacterial pathogens, such as *Bacillus anthracis* [80] and *Yersinia pestis* [81] were reported to have closed pangenomes. Rouli et al. [80] proposed that the pangenome’s nature reflects the organism’s lifestyle, and species that have an open pangenome can thrive in human and animal guts. However, it is uncertain if this applies to *C. baratii* as it does not colonize temporarily the gut microbiota and it has been found in several environments. It should be noted that this pangenome analysis might be limited due to the low number of *C. baratii* genomes available in the databases.

The pangenomic variation of the *C. baratii* species studied here could be driven by horizontal gene transfer (HGT) events, as suggested by the high number of genes associated with transposases, integrases, and phages (“L” COG category). This high unusual number could explain a high potential for gene gain or loss events (Figure 3). Interestingly, genes involved in defense mechanisms, recombination, and repair processes were encoded at a higher percentage within the accessory genes [82,83,84]. This also suggests variation in the genomic adaptations to stress among *C. baratii* genomes, which in addition, correlates with the variations found in AMR genes in this species. In contrast, genes encoding functions related to metabolism (i.e., amino acid, carbohydrates, butyrate production) were more represented within the core pangenome, indicating that these functions are conserved among *C. baratii* species.

To understand the pathogenicity of *C. baratii* and other pathogen clostridia described in Kiu et al. [7], we evaluated the toxins present in these genomes. Figure 5 shows that alpha-toxin plc (62.8% identity on average with plc from *C. perfringens,* Supplementary Table S3) is present in all *C. baratii* strains. The phospholipase C (plc) is a hydrolase involved in signal transduction processes [85]. Bacterial plc interacts with eukaryotic cell membranes and hydrolyzes phosphatidylcholine and sphingomyelin, causing cell lysis [86], and under certain thresholds oxidative stress [87] and gas gangrene [88]. We found evidence of the presence of virulence factors well conserved in *C. baratii* genomes. And we found only two *C. baratii* strains with complete plasmids (plasmid pNPD11_1 in CDC51267 and plasmid pCBJ in Sullivan strains). Additionally, we only found proteins from a plasmid (plasmid pNPD11_1) in strains that have the BoNT cluster (693-15, 694-15, 695-15, 771-14, and 796-15). This is relevant because these are the only genomes that are closed, the other strains have a draft genome. Knowing how they were acquired is a big challenge because is necessary to have more information on these strains and ideally have closed the genomes instead of what is available today. This bioinformatics prediction correlates with the β-hemolytic activity of *C. baratii* C3. Finally, predicted AMR results should be taken with caution, and should be validated experimentally. For example, CARD predicted fluoroquinolone resistance in *C. baratii* C3 through *gyrB* mutations, however, this microorganism was sensitive to ciprofloxacin. Apparently, C3 is resistant to tetracycline and rifampin, but we were not able to confirm this estimation. *Clostridium* species appear to be resistant in great numbers to sulfonamides.

The BoNT/F gene cluster was present in seven out of fifteen strains (*C. baratii* 693-15, 694-15, 695-15, 771-14, 796-15, Sullivan, and CDC51267 strains). Considering the clinical importance of this microorganism and that most studies reporting genomic information about this taxa are related to botulinum outbreaks, the proportion of *C. baratii* genomes with BoNT production potential is unclear and could be overlooked. The phylogeny of BoNT genes (Supplementary Figure S3) in *C. baratii* and *C. botulinum* strains showed a monophyletic lineage, indicating that the amino acid sequence is well conserved between these species. These findings agree with the high degree of conservation of the active site of the BoNT protein. In addition, BoNT sequences within *C. baratii* strains analyzed in this study, have a lack of genetic variation among BoNT sequences when compared to other *Clostridium* species and its presence in seven of fifteen genomes suggest a recent horizontal gene transfer event that is also supported by a large number of prophage proteins. That *C. baratii* strains acquired the BoNT cluster by HGT was proposed before based on the evidence of the presence of the same toxins in different groups of bacterial species [20] and that the genomic architecture showed that the BoNT cluster is flanked by two IS1182 gene copies [14,20].

The percentage of identity between the BoNT type F of *C. botulinum* and *C. baratii* strains was on average 72%, and the active site HEXXH was conserved between proteins of both species (Figure 8). We found a seven amino acid region upstream of the active site appeared to be missing across *C. baratii* genomes. However, this gap is not likely to impact the toxicity of BoNT because these amino acids do not interact with the molecular target of BoNT, the VAMP2 protein [71]. It is relevant to consider that the BoNT/F7 of *C. baratii* needs to recognize a longer peptide of the VAMP sequence to achieve cleavage [89] when identifying the toxin. In addition, there is a 30% difference in identity with other F serotypes of *C. botulinum.*

## 5. Conclusions

*C. baratii* is an understudied clostridial species that could pose a potential threat to human health considering some isolates could produce the botulinum toxin. Our comparative genomic analysis of fifteen genomes of *C. baratii* indicated a heterogeneous open pangenome and diversity in adaptation to stress processes. Certain *C. baratii* species seem to be resistant to tetracycline, the majority of *C. baratii* strains were equipped with phospholipase C. Seven out of fifteen genomes carried a complete BoNT/F7 cluster that is highly conserved among *C. baratii* genomes and displayed distinct differences compared to *C. botulinum* BoNT. The analysis we report helps to understand the properties of rare clostridial taxa and their understudied clinical relevance, highlighting their toxin-producing capabilities and likely resistance and adaptation mechanisms. 

## Data Availability

The genome of *C. baratii* strain C3 are available on https://www.ncbi.nlm.nih.gov/bioproject/PRJNA716081/ accessed on 11 November 2021.

## Supplementary Materials

The following are available online at https://www.mdpi.com/article/10.3390/microorganisms10020213/s1. Figure S1. *C. baratii* pan-genome computational statistics. Figure S1. Conserved and unique genes in *C. baratii* pangenome. Figure S2. Percentage of all CAZy categories identified in *C. baratii* pangenomes. Figure S3. BoNT phylogeny between *C. botulinum* and *C. baratii*. Figure S4. BoNT three-dimensional structure. Table S1. List of *Clostridium baratii* strains genomes from NCBI database [90,91,92,93,94,95,96,97,98,99,100,101]. Table S2. Genomes of pathogenic clostridia. List of pathogen *Clostridium* genomes used. Table S3. plc protein identity. % identity based on amino acid sequence identity (using blastp) with plc from *C. perfringens* as reference. Table S4. Prophages found in *C. baratii* strains. On a separate file. Table S5. Quantity of proteins from plasmids found in *C. baratii* strains.
