# Supplementary material for: Comparative Genomics of Clostridium baratii Reveals Strain-Level Diversity in Toxin Abundance
Source: Microorganisms. 2022 Jan 20;10(2):213. doi: 10.3390/microorganisms10020213 (PMC8879937; doi:10.3390/microorganisms10020213)
Supplement: Supplementary file 1 [file microorganisms-10-00213-s001.zip › microorganisms-1414802 -Supplementary Materials.pdf]

Supplementary Materials

Table S1. List of *Clostridium baratii* strains genomes from NCBI database.

Organism	Isolation Source	Type	Bioproject	Genome Size (bp)	Proteins	%GC	Collection Date	Geographic Location
Clostridium baratii MGYG-HGUT-00064	Human gut	metagenome	PRJEB33885	3,141,705	3078	28.1	2019	United Kingdom
Clostridium baratii 2789STDY5834956	Human fecal samples	metagenome	PRJEB10915	3,086,202	2930	28	2013	United Kingdom
Clostridium baratii 2789STDY5834907	Human fecal samples	metagenome	PRJEB10915	3,141,705	3028	28.1	2013	United Kingdom
Clostridium baratii Sullivan	Human stool (adult)	monoisolate	PRJNA225788	3,338,630	3169	28.26	2007	USA: NY
Clostridium baratii 771-14	Human stool	monoisolate	PRJNA275411	3,173,174	2839	28	2015	France
Clostridium baratii XCM	Soil	monoisolate	PRJNA290832	3,087,740	2855	28.1	2014	China: Shanghai
Clostridium baratii 695-15	Stool from botulism patient	monoisolate	PRJNA343447	3,057,605	2822	28	2015	France
Clostridium baratii 693-15	Stool from botulism patient	monoisolate	PRJNA343441	3,058,247	2818	28	2015	France
Clostridium baratii 796-15	Beef Meat	monoisolate	PRJNA315217	3,056,346	2802	28	2015	France
Clostridium baratii 694-15	Stool from botulism patient	monoisolate	PRJNA343444	3,059,409	2821	28	2015	France
Clostridium baratii CDC51267	Not Available	monoisolate	PRJNA301987	3,211,717	2935	28.28	Not Available	Not Available
Clostridium baratii MCC332	Human feces	metagenome	PRJNA548918	2,968,693	2843	28.1	2019	Ireland
Clostridium baratii L3_128_029G1	Infant feces	metagenome	PRJNA698986	3,015,344	2968	28.1	2016/2019	USA: Pittsburgh
Clostridium baratii L2_013_037G1	Infant feces	metagenome	PRJNA698986	3,041,624	2858	28	2016/2019	USA: Pittsburgh
Clostridium baratii C3	Human fecal	monoisolate	PRJNA716081	3,119,424	2945	28.2	2018	Chile: Santiago
Clostridium baratii 2789STDY5834956	Human fecal samples	metagenome	PRJEB10915	3,086,202	2930	28	2013	United Kingdom
Clostridium baratii 2789STDY5834907	Human fecal samples	metagenome	PRJEB10915	3,141,705	3028	28.1	2013	United Kingdom

Table S2. List of pathogen *Clostridium* genomes used.

Organism	Toxin	Disease
Clostridium botulinum ATCC3502	BoNT/A	Botulism [5,6]
Clostridium butyricum	BoNT/E [90]	Botulism [5,6]
Clostridium cadaveris AGR2141	*toxA*	Bacteraemia [91]
Clostridium carnis	-	Septicaemia [92]
Clostridium bifermentans ATCC638	A putative nagH (Mu-toxin), α-toxin (plc), colA, and pfoA (perfringolysin O) [93]	Bacteremia [93] and Empyema [94]
Clostridium celatum DSM1785	-	Infection [95]
Clostridium chauvoei JF4335	Necrotozin, d-hemolysin, a-hemolysin, b-deoxyribonuclease, and g-hyaluronidase	Animal infection [96]
Clostridium difficile 630	ftcdA, tcdB, and tcdR	gastrointestinal illness [97]
Clostridium paraputrificum AGR2156	ToxA, toxZ, epsF, and *nagH* (a mu-toxin).	Bacteremia and myonecrosis [98]
Clostridium novyi NT	-	Myonecrosis [96]
Clostridium fallax	-	Gastrointestinal diseases and bacteremia [99]
Clostridium perfringens ATCC13124	α-clostripain (ccp), α-toxin (plc), sialidase (nanI, nanJ, and nanH), tpeL peptidase, and colA	human food poisoning [7], and, gastroenteritis [4]
Clostridium septicum	a-lethal, necrotic, hemolytic; b-deoxyribonuclease; g-hyaluronidase; d-O2-labile hemolysin; and collagenase	neutropenic enterocolitis and gastrointestinal diseases [96]
Clostridium sordellii	TcsH and TcsL	Gastrointestinal diseases [100]
Clostridium sporogenes	-	Bacteremia [101]
Clostridium tertium	*nagH* (a mu-toxin).	Bacteremia [98]
Clostridium tetani E88	TeNT	Tetanus [3,96]

Table S3. % identity based on amino acid sequence identity (using blastp) with plc from *C. perfringens* as reference.

Organism	% Identity	% Coverage
Clostridium baratii MGYG-HGUT-00064	63.568	100
Clostridium baratii 2789STDY5834956	62.060	100
Clostridium baratii 2789STDY5834907	63.568	100
Clostridium baratii Sullivan	63.568	100
Clostridium baratii 771-14	63.317	100
Clostridium baratii XCM	63.317	100
Clostridium baratii 695-15	61.809	100
Clostridium baratii 693-15	61.809	100
Clostridium baratii 796-15	61.809	100
Clostridium baratii 694-15	61.809	100
Clostridium baratii CDC51267	63.065	100
Clostridium baratii MCC332	63.317	100
Clostridium baratii L3_128_029G1	63.317	100
Clostridium baratii L2_013_037G1	63.568	100
Clostridium baratii C3	62.563	100

Supplementary Table S4. Prophages found in *C. baratii* strains. On a separate file.

Supplementary Table S5. Quantity of proteins from plasmids found in *C. baratii* strains

Strain	Plasmid Protein	Plasmid Origin
Clostridium baratii 693-15	13/119	pNPD11_1
Clostridium baratii 796-15	13/119	pNPD11_1
Clostridium baratii 694-15	13/119	pNPD11_1
Clostridium baratii CDC51267	119/119	pNPD11_1
Clostridium baratii 695-15	13/119	pNPD11_1
Clostridium baratii Sullivan	212/212	pCBJ
Clostridium baratii 771-14	103/119	pNPD11_1

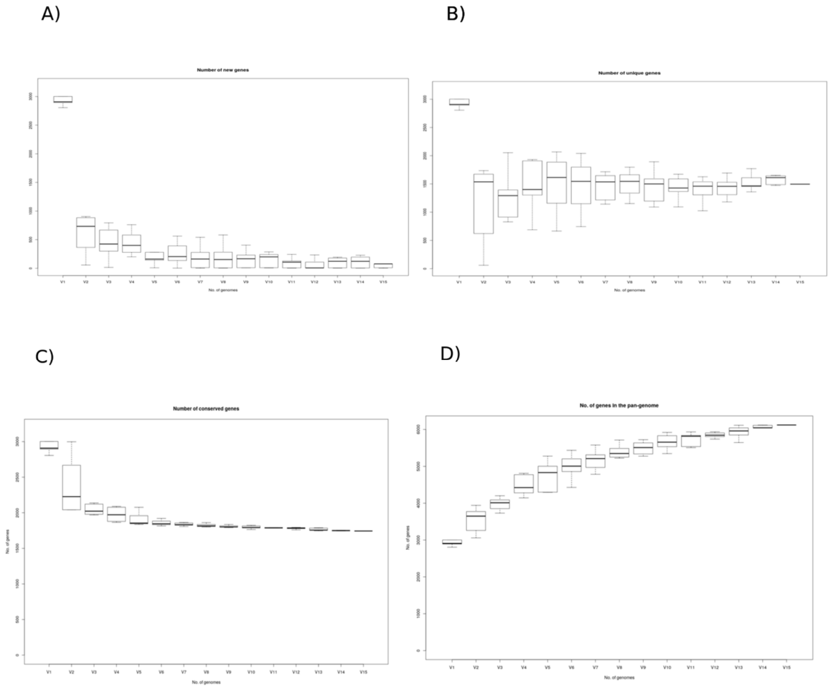

Figure S1. *C. baratii* pan-genome computational statistics. (A) Box plot of new genes added to the pan-genome iteration per additional strain. New genes do not converge to zero upon addition of genome at n = 15, an average of 226 genes are contributed to the gene repertoire. **(B)** Calculation of unique gene addition. **(C)** Calculation of conserved genes per genome addition to the gene pool. **(D)** Number of genes in the pan-genome over 15 genome additions.

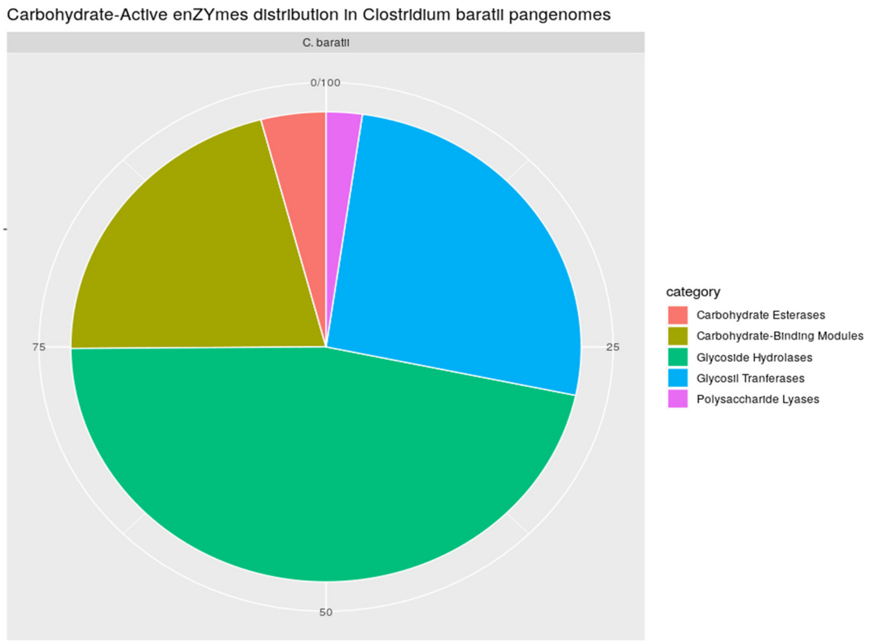

Figure S2. Percentage of all CAZy categories identified in *C. baratii* pangenomes. CAZy category definitions are as follows: GH, glycoside hydrolases; GT, glycosyltransferases; CBM, carbohydrate-binding modules; CE, carbohydrate esterases, and PL, polysaccharide lyases.

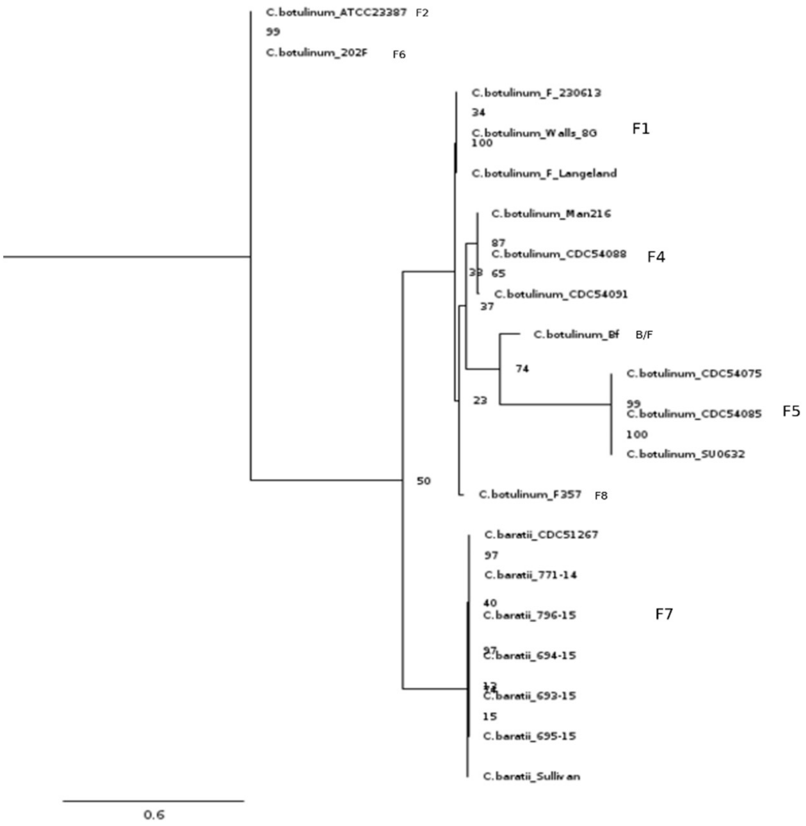

Figure S3. Phylogeny of BoNT gene in *C. baratii* and *C. botulinum* strains.

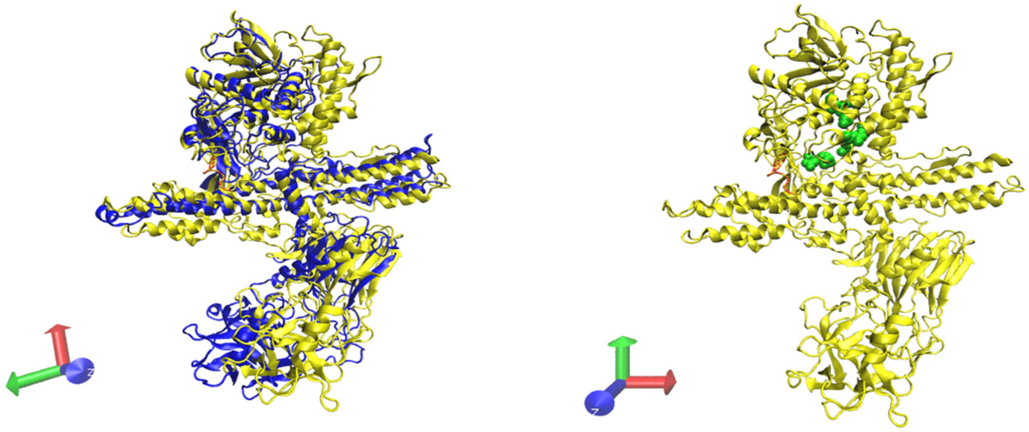

(**A**) (**B**)

Figure S4. (**A**) Three-dimensional structure alignment between BoNT/A1 (PDB:3BTA in yellow) and three-dimensional structure of BoNT/F7 of *C. baratii* (blue) predicted with Phyre2. In red the aa lost in BoNT/F7. (**B**) Three-dimensional structure of BoNT/A1 in yellow; in red aa lost in BoNT/F7; in green aa of active site.
